# Mapping of the benzoate metabolism by human gut microbiome indicates food-derived metagenome evolution

**DOI:** 10.1038/s41598-021-84964-6

**Published:** 2021-03-10

**Authors:** Monika Yadav, Avinash Lomash, Seema Kapoor, Rajesh Pandey, Nar Singh Chauhan

**Affiliations:** 1 Department of Biochemistry, Maharshi Dayanand University, Rohtak, Haryana 124001 India; 2 Pediatrics Research and Genetic Laboratory, Maulana Azad Medical College, New Delhi, 110002 India; 3 Genomics and Molecular Medicine, CSIR-Institute of Genomics and Integrative Biology (CSIR-IGIB), Delhi, 110007 India

**Keywords:** Metagenomics, Microbiome, Bacterial genes, Bacteriology

## Abstract

Sodium benzoate is one of the widely used food preservatives and its metabolism in the human body has been studied only with the host perspective. Despite the human gut microbiome being considered as a virtual human organ, its role in benzoate metabolism is yet to be elucidated. The current study uses a multi-omic approach to rationalize the role of human gut microbes in benzoate metabolism. Microbial diversity analysis with multiple features synchronously indicates the dominance of Bacteroidetes followed by Firmicutes, Actinobacteria, and Proteobacteria. Metagenomic exploration highlights the presence of benzoate catabolic protein features. These features were mapped on to the aerobic and anaerobic pathways of benzoate catabolism. Benzoate catabolism assays identified statistically significant metabolites (*P* < 0.05) associated with the protocatechuate branch of the beta-ketoadipate pathway of the benzoate metabolism. Analysis of the 201 human gut metagenomic datasets across diverse populations indicates the omnipresence of these features. Enrichment of the benzoate catabolic protein features in human gut microbes rationalizes their role in benzoate catabolism, as well as indicates food-derived microbiome evolution.

## Introduction

Sodium benzoate being a safe antimicrobial agent is commonly used as a food preservative. The human intestine receives an average of 0–5 mg kg^−1^ of benzoate per day either as a food additive^1^ or as a metabolic end-product of polyphenol diets^2^. Metabolism of the sodium benzoate is well described in humans^1^. Almost all the absorbed sodium benzoate is excreted out of the human body through the urogenital system as amino acids adduct i.e. hippuric acid^3,4^. The human metabolic framework is considered as a sole element in sodium benzoate detoxification^4^. From the last few decades, microbes have been identified as a key human companion and considered as a virtual organ of the human system^5^. Human microbial companion known as the human microbiome resides in and on the human body^6^ and plays a vital role in the various physiological processes^5,6^ that cumulatively allows the host to sustain healthy physiology^6^. Even microbiome dysbiosis either due to any genetic^7^ or epigenetic changes^8^ results in the onset of various physiological^5,9^ and metabolic disorders^10^.

Benzoate is a well-proven antimicrobial agent^11^, however, its limited availability in the human gut might have failed to induce any antimicrobial effect on gut microbes. Despite limited concentration, antimicrobial food additives were found to induce gut metagenome enrichment for their catabolism^12^. Likewise, continuous dietary benzoate exposure might have allowed the human gut microbes to evolve benzoate catabolic machinery. However, to date, no efforts have been made to explore the benzoate catabolic machinery of the human gut microbes. Simultaneously, sodium benzoate metabolic studies in various primates also strongly indicate the possibility of an alternate mechanism in addition to the glycine conjugation^4,5^. Additionally, free-living microbes like *Pseudomonas putida*^13^, *Rhodopseudomonas palustris*^14^, *Thauera aromatica*^15^, *Azoarcus* *sp*.^16^, *Rhodococcus sp.*^17^, etc.,^18–21^ were characterized for benzoate catabolic potential. These microbes were identified to catabolize benzoate either with aerobic or anaerobic mechanisms^13–21^. Gene products for these pathways were identified from gram-positive, as well as gram-negative microorganisms, indicating their invariable presence within the microbial clade^13–21^. All this information generates a strong possibility for the presence of efficient catabolic machinery among human gut microbes to counter the antimicrobial effect of the sodium benzoate. As there is a paucity of information, the current study has been designed with a culture-independent approach to explore the human gut microbiome for its structure, genetic makeover, as well as its metabolic machinery involved in benzoate catabolism. The outcome of this study would enhance our understanding of human microbiome functions, defining essential features for microbiome establishment, sustenance, and colonization. This information could be harnessed to take forward the concept of microbiome therapeutics for human healthcare.

## Results

### Metagenomic analysis of human gut microbiome

A total of 40,273,834 sequences totaling 12,068,464,866 basepairs with an average length of 300bps were obtained after paired-end sequencing chemistry with MiSeq System (Illumina, USA) (Supplementary Table S1).

### Human gut microbiome composition based on metagenomic RNA and Protein features

**Figure 1:**
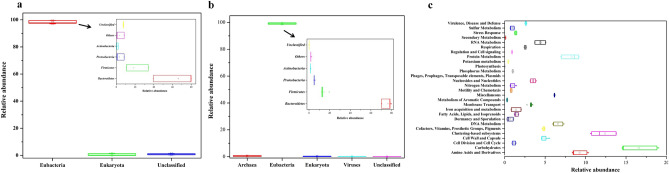
Taxonomic and functional characterization of the human gut metagenome. Microbiome composition based on rRNA features (**a**) and protein features (**b**). Subsystem database clustering of the protein features based on their probable functional potential subsystem database (**c**). Taxonomic distribution of eubacterial specific rRNA features and protein features at phylum level is shown in the inset (**a**,**b**).

Quality filtered metagenomic sequences have a representation of ribosomal RNA features within 245,344 sequences, while 6,868,080 sequences (56.86%) contain predicted proteins with known functions, and 5,208,955 sequences (43.13%) contain predicted proteins with unknown functions (Supplementary Table S1). The majority of the ribosomal features (98.41 ± 1.18%) shared their taxonomic affiliation within the eubacterial clade, while a very small fraction (0.68 ± 0.7%) were identified to share their homology within Eukaryota domain in addition to 0.9 ± 0.48% unclassified RNA features (Fig. 1a). Likewise, Phylogenetic analysis based on RNA features, the majority of identified protein features (99.22 ± 0.51%) shared homology with the protein affiliated within the eubacterial domain followed by Archaea (0.48 ± 0.35%), Eukaryota (0.23 ± 0.13%), Viruses (0.06 ± 0.03%), and others (0.0015 ± 0.001%) (Fig. 1b).

### Metabolic potential of the human gut microbes

A plethora of microbial companions in the human gut play a variety of functions for their survival and maintenance within the human gut^5,6^. Protein features of the human gut metagenome were used to create the annotation profile to define their probable functional categories in the subsystem database. Subsystem-based categorization indicated more than half (52.91%) of the total identified protein features associated with general cellular metabolism (carbohydrate-, protein-, amino acids-, nucleic acid- metabolism). Most surprisingly, a limited number of protein features (1.37 ± 0.11%) are involved in microbial stress response physiology (dormancy & sporulation, stress response), while an elevated number of protein features (5.65 ± 0.75%) are associated with virulence and genetic transformations (Fig. 1c). The human gut is comparatively more stable than its environment counterpart which could be limiting the requirements of stress response features.

### Benzoate metabolic features in the human gut metagenome

Metagenomic exploration of the human gut microbiome has identified genetic features associated with anaerobic (Benzoyl-CoA degradation pathway) and aerobic (oxygenase-coupled central aromatic intermediate metabolic pathway) (Table 1). These genetic features were predominantly dispersed among various microbial groups like Proteobacteria, Firmicutes, Bacteroidetes, and Actinobacteria (Supplementary Fig. S1, S2, and S3).

**Table 1:** Human gut microbiome protein features mapped for sodium benzoate catabolism.

Benzoate catabolic pathway	Protein features	Positive hits
**Anaerobic benzoate metabolism**		
Anaerobic benzoate metabolism	Benzoyl-CoA reductase subunit BadD (EC 1.3.99.15)	18
	Benzoyl-CoA reductase subunit BadE (EC 1.3.99.15)	88
	Benzoyl-CoA reductase subunit BadF (EC 1.3.99.15)	15
	Benzoyl-CoA reductase subunit BadG (EC 1.3.99.15)	386
	2-hydroxycyclohexanecarboxyl-CoA dehydrogenase, BadH	11
	Glutaryl-CoA dehydrogenase (EC 1.3.99.7)	3
	3-hydroxybutyryl-CoA dehydrogenase (EC 1.1.1.157)	4
	Acetyl-CoA acetyltransferase (EC 2.3.1.9)	115
**Aerobic benzoate metabolism**		
Catechol mediated *ortho* cleavage pathway	Benzoate 1,2-dioxygenase, BenA (EC 1.14.12.10)	4
	Catechol 1,2-dioxygenase, CatA (EC 1.13.11.1)	7
	Muconate cycloisomerase, CatB (EC 5.5.1.1)	13
	Muconolactone isomerase, CatC (EC 5.3.3.4)	1
Protocatechuate mediated *beta*-ketoadipate pathway	4-hydroxybenzoate 3-monooxygenase (EC 1.14.13.2)	17
	Protocatechuate 3,4-dioxygenase alpha chain, PcaG (EC 1.13.11.3)	4
	Protocatechuate 3,4-dioxygenase beta chain, PcaH (EC 1.13.11.3)	12
	3-carboxy-cis,cis-muconate cycloisomerase, PcaB (EC 5.5.1.2)	11
	4-carboxymuconolactone decarboxylase, PcaC (EC 4.1.1.44)	553
	Beta-ketoadipate enol-lactone hydrolase, PcaD (EC 3.1.1.24) or 3-oxoadipate enol-lactonase, PcaD (EC 3.1.1.24)	2
	3-oxoadipate CoA-transferase subunit A, PcaI (EC 2.8.3.6)	7
	3-oxoadipate CoA-transferase subunit B, PcaJ (EC 2.8.3.6)	1
	Beta-ketoadipyl CoA thiolase, PcaF (EC 2.3.1.16)	5
	Pca operon regulatory protein, PcaR	19
	Succinyl-CoA:3-ketoacid-coenzyme A transferase subunit A, ScoA (EC 2.8.3.5)	2
**Benzoate Transporters**	Benzoate MFS transporter, BenK	26
	4-hydroxybenzoate transporter, PcaK	15

### Metabolic features for anaerobic benzoate metabolism

**Figure 2:**
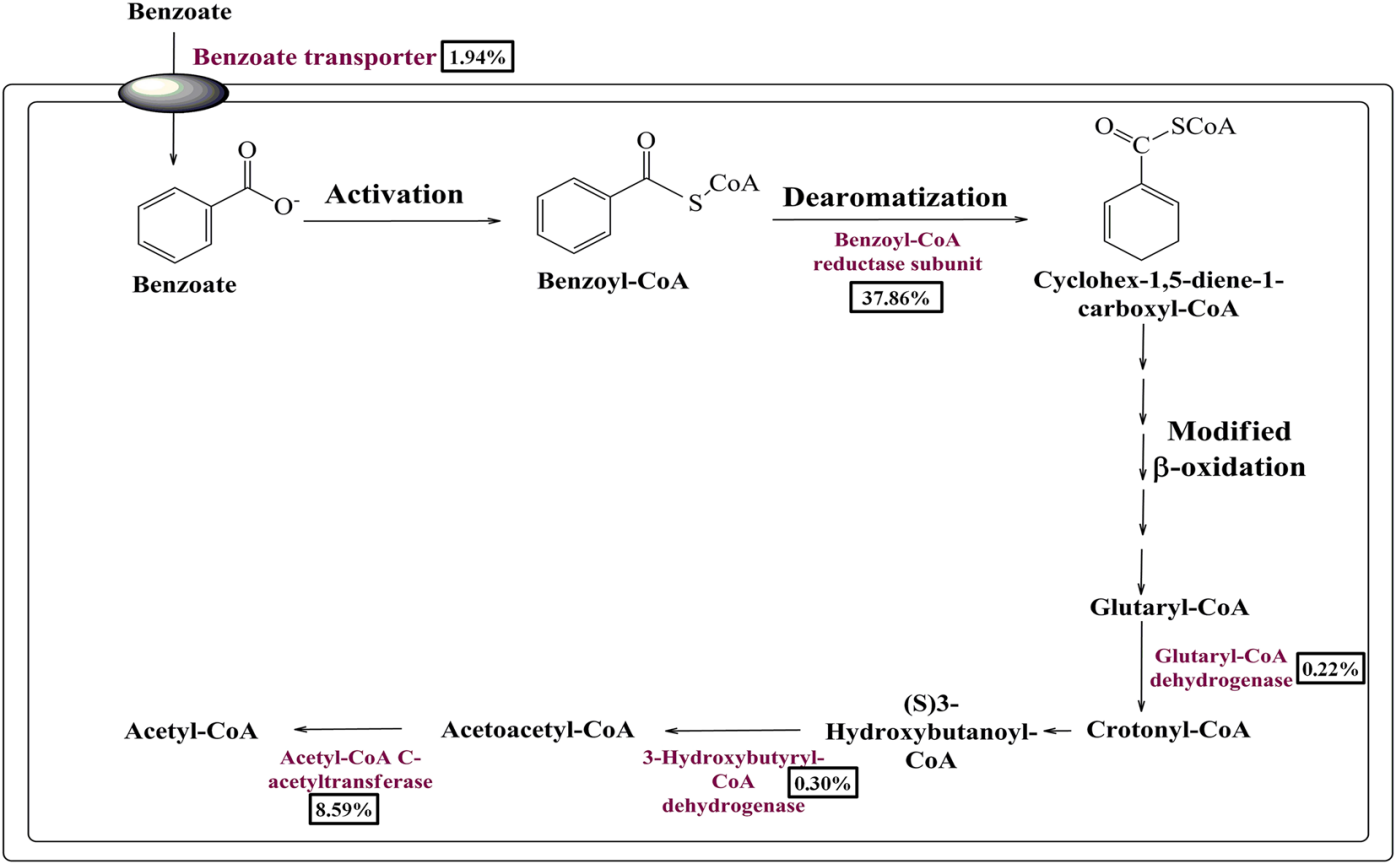
The delineated anaerobic pathways of benzoate metabolism in the human gut microbiome. The relative abundance of the benzoate catabolic protein features in the current metagenome datasets is shown in percentage.

The anaerobic benzoyl-CoA degradation pathway is a predominantly multistep process. Initially, benzoate is converted into benzoyl CoA in the activation process followed by its conversion into Aliphatic C7 dicarboxyl CoA by the dearomatization and β-oxidation in the upper pathway^18–21^. Aliphatic C7 dicarboxyl CoA is further converted into acetyl CoA and CO_2_ in lower pathway^18–21^. Denitrifying bacteria like *Thauera aromatica*, *Azoarcus sp*. and strict anaerobic Fe(III)-reducing *Geobacter metallireducens*, *Syntrophus aciditrophicus* employ this pathway to utilize benzoate to meet energy demands^18^. Exploration of the human gut metagenome identifies genes encoding benzoyl-CoA reductase (BadD, BadE, BadF, BadG, and BadH) with the potential to catalyze benzoyl CoA dearomatization. Glutaryl-CoA dehydrogenase, 3-hydroxybutyryl-CoA dehydrogenase, and acetyl-CoA acetyltransferase associated with a lower pathway of anaerobic benzoate metabolism (Table 1, Fig. 2). Surprisingly, there was not a single hit for benzoate CoA ligase in the studied metagenome. The absence of this enzyme generates a probability that either this function is being carried out by a novel enzyme or these pathways are inactive. Phylogenetic affiliation analysis indicates that these protein features were predominantly affiliated with Firmicutes (87.5%) followed by Proteobacteria (8.86%), Actinobacteria (0.73%), and unclassified bacteria (2.91%) (Supplementary Fig. S1). Within Firmicutes, the majority of protein features were affiliated to the *Ruminococcaceae* (29.7%), *Lachnospiraceae* (14.5%), and *Veillonellaceae* (9.14%) (Supplementary Fig. S1).

### Metabolic features for aerobic benzoate metabolism

**Figure 3:**
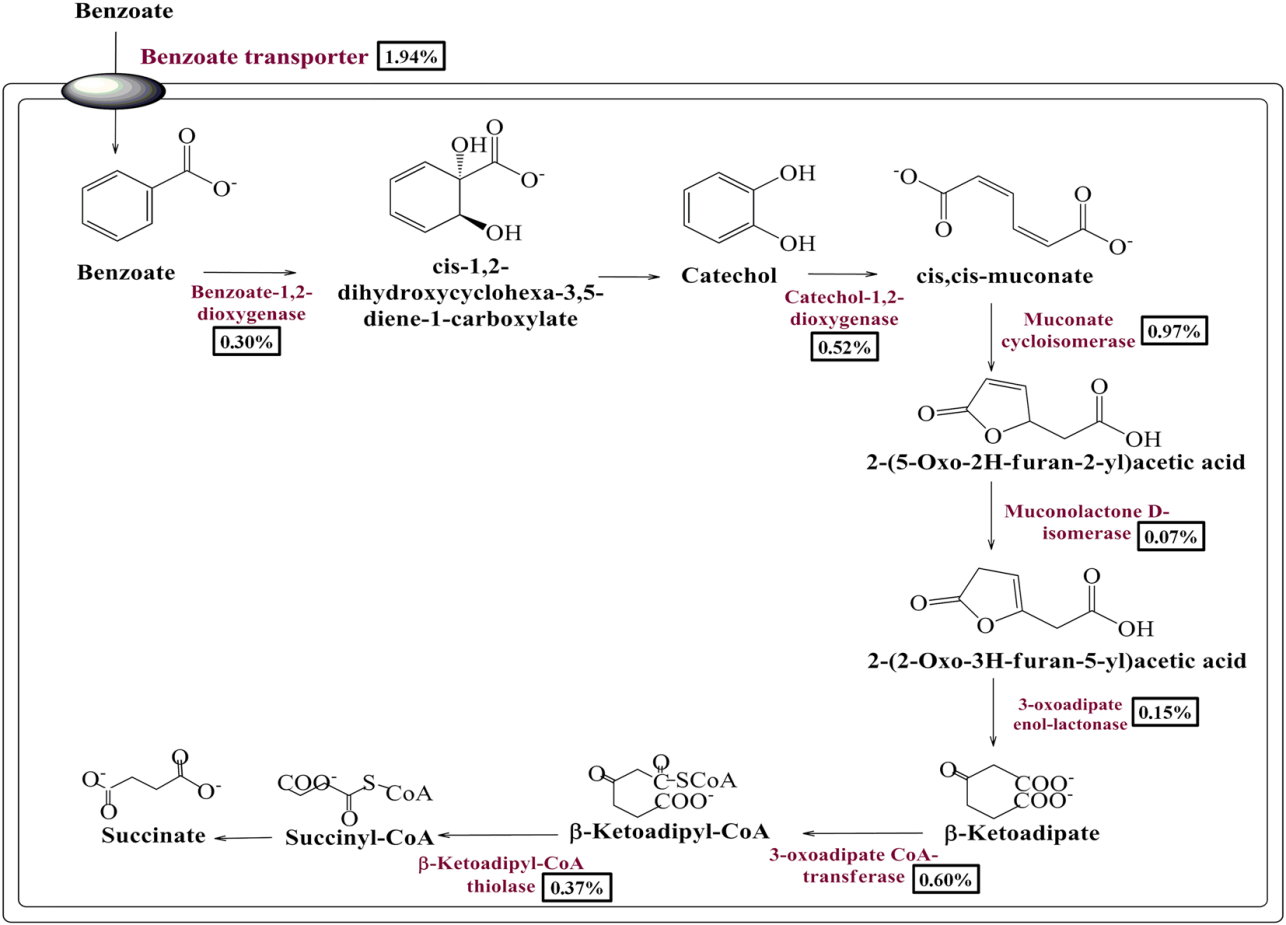
Mapping of the metabolic features associated with catechol mediated *ortho*-cleavage pathway of benzoate metabolism. The relative abundance of the benzoate catabolic protein features in the current metagenome datasets is shown in percentage.

The aerobic benzoate metabolic pathway involves an oxygenase-dependent upper pathway converting benzoate into central aromatic intermediates like catechol or protocatechuate^19,22^. These central aromatic intermediates were catabolized into TCA cycle intermediates in a lower metabolic step which involves dearomatization ring cleavage of central intermediates^19–22^. Metagenomic exploration identified protein features for cellular uptake of benzoate (BenK) or benzoate derivatives (PcaK) and catalyzing upper and lower pathway of aerobic benzoate metabolism (Table 1). Benzoate-1,2-dioxygenase (BenA), 4 hydroxybenzoate-3-monooxygenase features of the current metagenomic dataset could catalyze the upper pathway reaction by converting benzoate into catechol and protocatechuate respectively. Catechol and protocatechuate are central aromatic intermediates that were catabolized by the enzymes associated with the convergent lower metabolic pathway^12,22^. Human gut metagenome indicates the presence of cat operon genes (*catA*, *catB*, *catC*) encoding proteins homologous to the catechol-1,2-dioxygenase (CatA), muconate cycloisomerase (CatB), and muconolactone D isomerase (CatC), respectively. These proteins were characterized in *Pseudomonas putida*^13,22^ for the *ortho* cleavage of catechol into 2- (2-oxo-3H-furan-5-yl) acetic acid (Fig. 3). The 2- (2-oxo-3H-furan-5-yl) acetic acid is metabolized into TCA cycle intermediates by the encoded proteins of *pcaD, pcaI/J*, and *pcaF* genes of the *pca* operon as a convergent metabolic pathway (Fig. 3). Proteins associated with benzoate catabolism via *ortho*-cleavage of catechol were phylogenetically affiliated with the proteins of Bacteroidetes/Chlorobi (62.78%), Proteobacteria (20.278%), Firmicutes (12.5%), Chloroflexi (0.278%), and unclassified bacteria (4.167%) (Supplementary Fig. S2).

**Figure 4:**
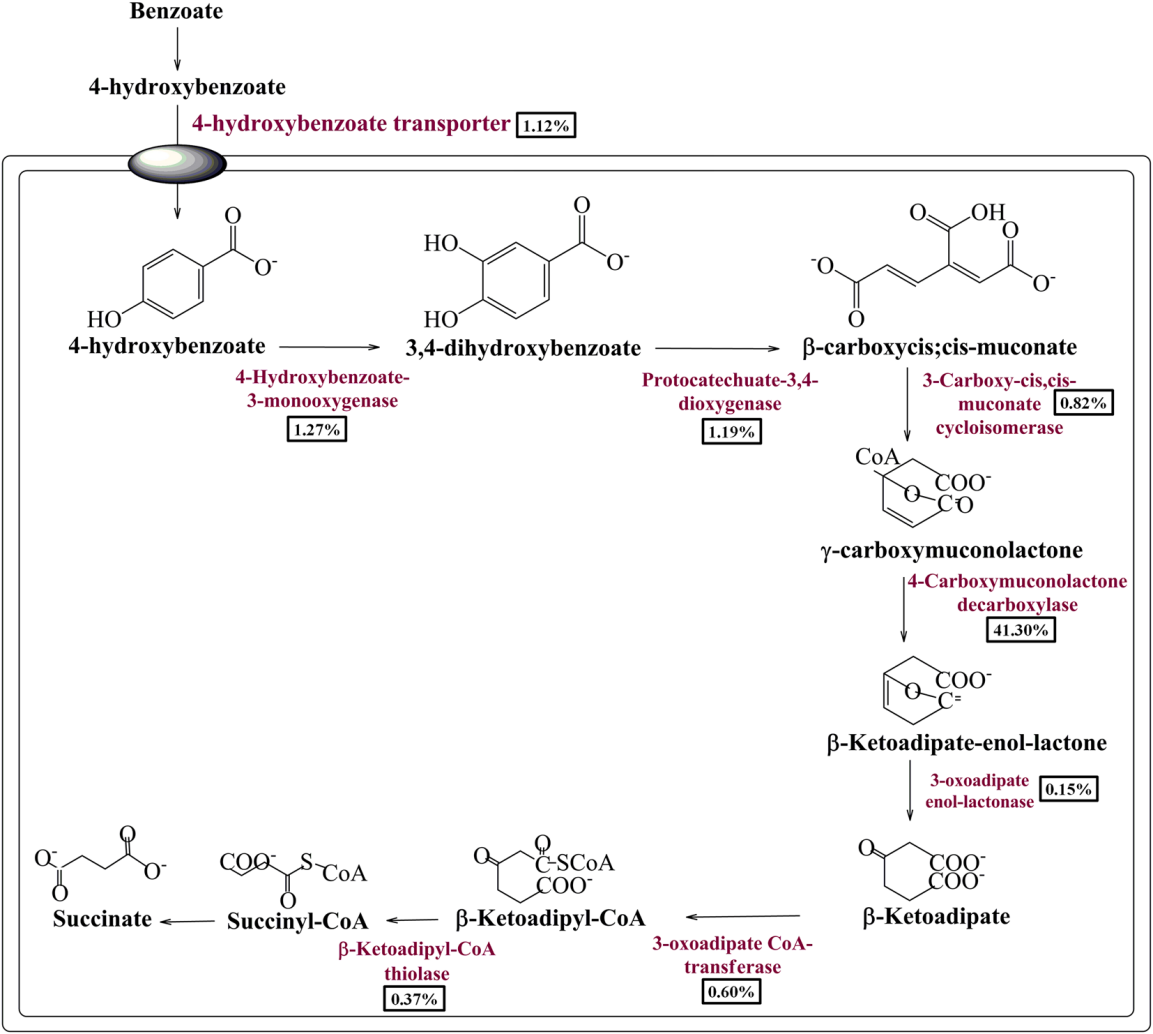
Mapping of the metabolic features associated with oxygenase coupled protocatechuate mediated aerobic pathway for benzoate metabolism. The relative abundance of the benzoate catabolic protein features in the current metagenome datasets is shown in percentage.

Manual curation of the current metagenomics dataset indicates several genes sharing homology with *pcaB*, *pcaC*, *pcaD*, *pcaG/H, pcaI, pcaJ, pcaK, pcaF*, and *pcaR* encoding proteins associated with catabolism of the protocatechuate into the TCA cycle intermediates^19–22^ (Table 1, Fig. 4). These genes encode *p*-hydroxybenzoate transporter (PcaK) for cellular transport of benzoate. The *p*-hydroxybenzoate can be sequentially metabolized by the PcaG/H, PcaB, PcaC, PcaD, PcaI/J, PcaF into succinyl CoA (Fig. 4). In addition to these protein features, the current dataset harbors PcaR to regulate the expression of the *pca* operon^23^. Proteins associated with the protocatechuate branch of the beta-ketoadipate pathway were phylogenetically affiliated with Firmicutes (44.75%), Bacteroidetes/Chlorobi (24.52%), Proteobacteria (18.46%), Verrucomicrobia (1.182%), Actinobacteria (0.88%), and unclassified bacteria (10.19%) (Supplementary Fig. S3).

### Human gut metagenome datasets of the diverse populations

**Figure 5:**
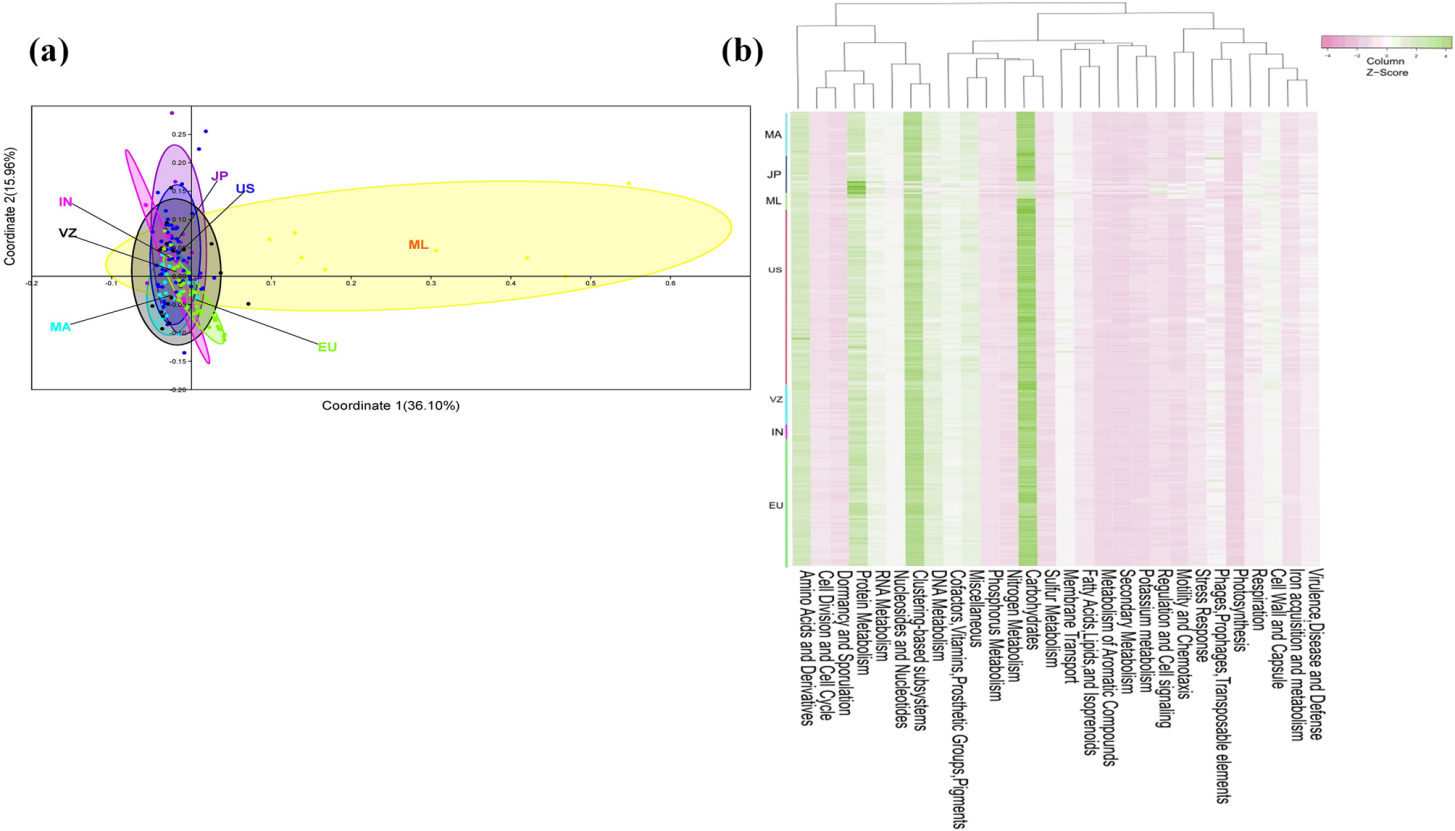
A comparison of the metabolic potential of the gut metagenomes across diversified populations. Principle coordinate analysis (PCoA) (**a**) and heatmap (**b**) showing the functional relationship and distribution of subsystem protein features among gut metagenomes of various populations (Malawi (MA), Malaysia (ML), Japan (JP), Europe (EU), USA (US), Venezuela (VZ), and India (IN)).

**Figure 6:**
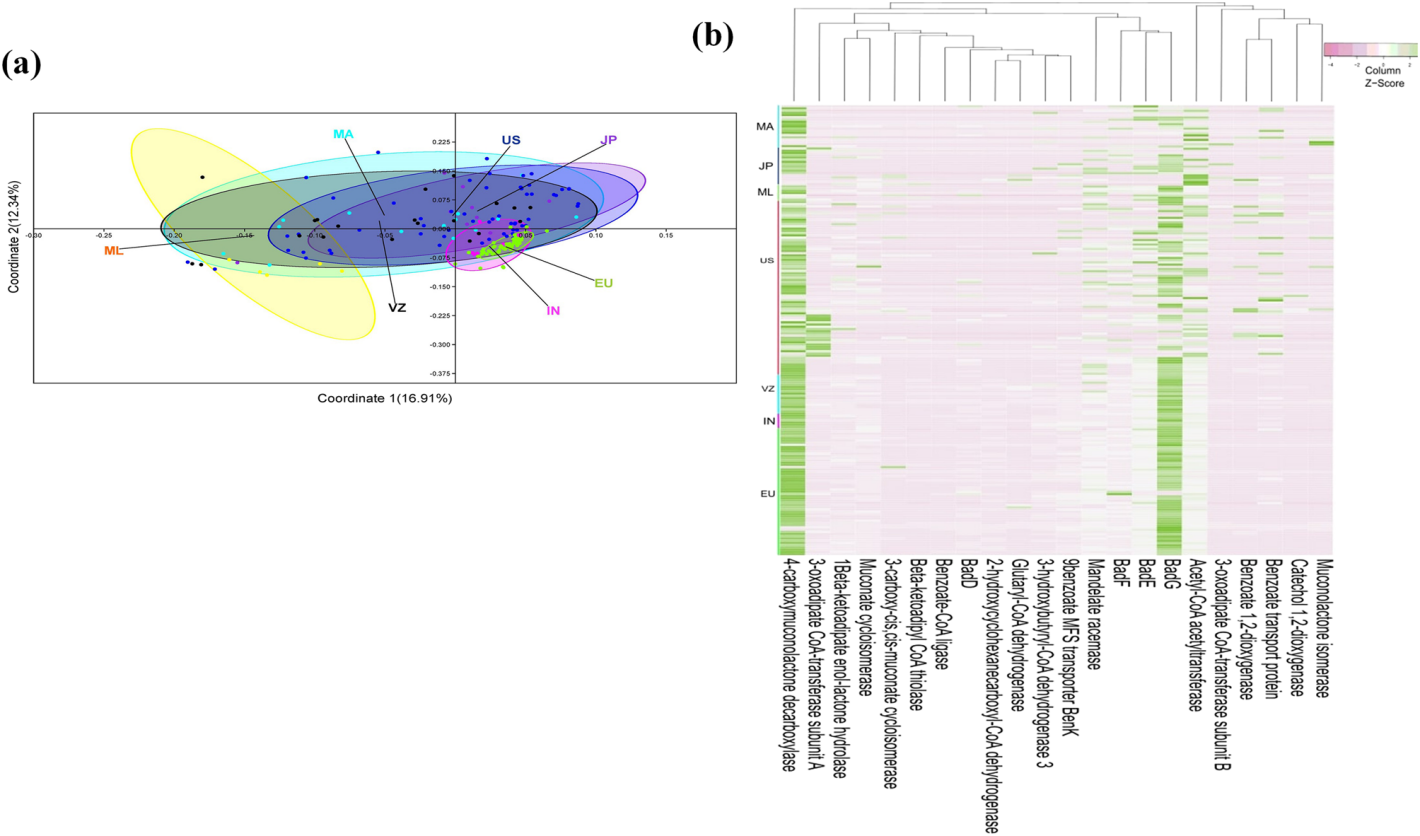
Benzoate catabolic protein features across the various gut metagenomes. Principal component analysis (**a**) and heatmap (**b**) showing the functional relationship and distribution of benzoate catabolic protein features among the gut metagenomes of various populations (Malawi (MA), Malaysia (ML) Japan (JP), Europe (EU), USA (US), Venezuela (VZ), and India (IN)).

Human gut metagenome datasets (n = 201) of diverse populations were downloaded (Supplementary Table S2) and analyzed for genetic and functional diversity to assess the similarity and uniqueness of the current metagenome datasets. Ribosomal features of the current metagenomic dataset showed a good correlation of (r > 0.99) at the domain level with ribosomal features identified in other metagenome datasets. The PERMANOVA test was used to assess how much the overall variation could be explained in the gut metagenomic datasets of the studied populations (Supplementary Table S3). Though no notable separation (*P* = 1.0) was observed among gut metagenome datasets of the individuals from India, Japan, Venezuela, and USA, however these metagenomes were found significantly distinct (*P* ≤ 0.05) from metagenome datasets of Malaysian, Malawi, and European populations. Similarly, metagenome datasets of Malaysian and Malawi population did not show any significant difference (*P* = 0.59). The metagenome dataset of the European population was remarkably distinct from other studied populations (*P* ≤ 0.05). Principal coordinate analysis (PCoA) analysis also made a similar observation (Fig. 5a). Heatmap analysis indicated the distribution pattern of the subsystem categorized protein features among gut metagenomes (Fig. 5b). Exploration of the gut metagenome datasets indicated the presence of the protein features associated with benzoate catabolism in each gut metagenome dataset (Supplementary Table S4). Principal coordinate analysis (PCoA) with benzoate catabolic protein features showed clustering of various populations except the Malaysian population (Fig. 6a). Despite the presence of benzoate catabolic protein features in every metagenome dataset, heatmap indicated their differential distribution among the gut metagenome datasets (Fig. 6b). The omnipresence of the benzoate catabolic protein features in studied gut metagenomes (Supplementary Table S4) clearly indicates the metabolic potential of the gut microbiome to catabolize benzoate, however, their functioning needs to be assessed through functional assays.

### Functional assay for sodium benzoate catabolism

Functional assessment of sodium benzoate catabolism was performed to identify functional benzoate metabolic machinery of the human gut microbiome. LC–MS profile assessment indicated that the human gut microbes have completely catabolized the available sodium benzoate within 30 min of incubation (Supplementary Fig. S4). Additionally, many metabolic features of the mass spectra were positively matched with several metabolites associated with benzoate catabolic pathways (Table 2). Identified metabolites were only found associated with aerobic protocatechuate intermediated benzoate metabolic pathway.

**Table 2:** List of statistically significant (p < 0.05) metabolites associated with benzoate catabolic pathways.

Metabolite	Benzoate catabolic pathway	Rf value	m/z value	Structure
Benzoate	Common in all	6.8213	160.9992	
4-Hydroxybenzoate	Protocatechuate branch of beta-ketoadipate pathway	17.76	216.9519	
Protocatechuate	Protocatechuate branch of beta-ketoadipate pathway	15.29	127.03	
Carboxy muconolactone	Protocatechuate branch of beta-ketoadipate pathway	15.93	201.003	
3-Carboxy-*cis,cis*-muconate	Protocatechuate branch of beta-ketoadipate pathway	17.29	201.0051	
Succinate	Protocatechuate & Catechol branch of β-ketoadipate pathway	0.81158	137.045	

## Discussion

Sodium benzoate possesses antibacterial properties, hereby, it is commonly used as an inexpensive preservative in the packaged food items^11^. Sodium benzoate enters the human system through enterocytes absorption and hepatic portal circulation. Humans are known to have biochemical machinery to metabolize and excrete out these xenobiotics as hippuric acid adducts^3,24^. Recently, various studies confirmed the role of the human gut microbiome in various metabolic processes like drug metabolism^25^, food digestion^5^, polyphenol metabolism^12,26^, antibiotic chemical modifications^27^, etc. Surprisingly, there is no information about the influence of the human gut microbiome on dietary sodium benzoate and vice-versa. Recently, a gut microbiome dynamics study was performed to explore the influence of sodium benzoate as an antimicrobial agent on the human gut microbiome composition^28^. This study confirmed the non-significant impact of sodium benzoate on human gut microbiome composition^28^. Additionally, there is a disparity between the amount of sodium benzoate intake and its excretion as hippuric acid^4^. It generates a strong possibility that there is an alternative unexplored metabolic pathway that consumes a fraction of sodium benzoate and does not allow its excretion as hippuric acid^24^. These cumulatively indicate that the human gut microbiome possibly plays a vital role in sodium benzoate catabolism either to protect themselves from its antimicrobial effects or to use it as an energy source. Hereby, the current study has been carried out finding functional genetic machinery involved in the bio-catabolism of the frequently used preservative sodium benzoate.

Shotgun sequenced metagenomic dataset captures complete diversity irrespective of taxonomic boundaries^29^, hence, the metagenomic dataset is helpful to draw a holistic and accurate picture of the studied microbiome. The human gut metagenome dataset was explored to define human gut microbiome composition based on rRNA gene features, as well as protein features. Microbiome composition deciphered with RNA & protein features of metagenomic dataset analysis reveals a similar outcome for eubacteria taxonomic affiliation. Bacteroidetes were uniformly dominant in both datasets followed by Firmicutes and Proteobacteria with slight variations in their percentage share. These results are in line with the various global microbiome studies^7,8^, and studies carried on Indian subjects^6^. The abundance of Bacteroidetes in the human gut microbiome could be due to their advantage in terms of possessing efficient catabolic machinery to harvest energy from complex carbohydrates^6^, which is a prime dietary component in north India. Metagenomic datasets also have a minor fraction of genetic and proteomic features affiliated with archaea, viruses, fungi, as well as with higher eukaryotes. These results are substantiating various studies that the human gut microbiome is a complex architecture having various live forms dominated by the eubacterial clade^6–9^.

Functional annotation and clustering of metagenomic protein features identified a good percentage of protein features associated with carbohydrate metabolism defining the enrichment of proteins involved in diversified sugar-utilization monosaccharides (Sorbitol, Gluconate, Glucouronides, etc.), oligosaccharides (Trehalose, Fructo-oligosaccharides, etc.), complex carbohydrates (cellulosome, xyloglucans, etc.,), sugar alcohols, and organic acids. The presence of a large array of proteins involved in diversified sugars’ metabolism to meet their energy requirements paves the way towards the successful commensalism of the human gut microbiome^30^. In addition to it, a plethora of proteins was identified to play a vital role in the cellular physiology and metabolism required for their growth and sustenance. More surprisingly, the human gut microbiome has a lower percentage of protein features associated with dormancy & sporulation, photosynthesis, cell signaling, in comparison to various environmental microbiomes^31^. In contrast, a slightly higher percentage of protein features associated with virulence, diseases & defense; Phages, Prophages, Transposable elements, Plasmids than environmental counterparts^31^. This variability could be seen as evolutionary adaptations to meet up environmental requirements for successful colonization^32^.

As an answer to the key question, many gene clusters/protein features potentially associated with benzoate metabolism were identified from the human gut metagenome dataset. Homologs of these putative protein features in various environmental microorganisms were well characterized for their functional role in benzoate catabolism^13–23^. Metagenomic exploration of the human gut microbiome has identified genetic features associated with anaerobic (Benzoyl-CoA degradation pathway)^18,20^ and aerobic (oxygenase coupled central aromatic intermediate metabolic pathway)^19–23^. In the environment, anaerobic microorganisms metabolize benzoate predominantly into TCA cycle intermediates through the anaerobic Benzoyl-CoA degradation pathway^18–20^. While aerobic micro-organisms predominantly utilize central aromatic intermediate metabolic pathway (either protocatechuate mediated beta-ketoadipate pathway or catechol mediated *ortho*/*meta*-cleavage pathway of benzoate oxidation^19–23^ and hybrid box pathway^18–20^ to catabolize benzoate into TCA cycle intermediates.

Identified protein features indicate the prevalence of major metabolic pathways of benzoate catabolism i.e. aerobic and anaerobic pathways^18–23^. The presence of these pathways indicates potentially-independent evolution of benzoate catabolic machinery among the human gut microbiome. This view is further supported by the outcome based on the phylogenetic affiliation of protein features associated with benzoate metabolism. Phylogenetic affiliation analysis of potential hits indicates the enrichment of these pathways in diversified microbial groups. Protein features in anaerobic benzoate metabolism pathways within Firmicutes followed by Proteobacteria and Actinobacteria. While protein features associated with the catechol branch of the central aromatic intermediate metabolic pathway got enriched within Bacteroidetes, followed by Proteobacteria and Firmicutes. Protein features of the Protocatechuate branch of the beta-ketoadipate pathway were equally distributed among Firmicutes, Bacteroidetes, and Proteobacteria. Except for selective enrichment, benzoate catabolic protein features showed phylogenetic affiliation with the proteins of the major gut microbial groups. It also indicates why there was a non-significant effect of sodium benzoate diet on human gut microbiome composition^28^. Comparative metagenomic analysis showed that despite the varied genetic and metabolic similarities, benzoate catabolic features were omnipresent among studied metagenomic datasets. It also indicates that despite the diverse ethnicity and food habits of the host, their inhabiting microbes have enriched themselves with benzoate catabolic potential. These results also strengthen the diet-driven gut microbiome evolutionary hypothesis^33^.

It was commonly observed that the conclusions and outcomes of various microbiome studies were drawn after analyzing the metagenomic dataset, which was based on one hypothesis i.e. if a gene for a defined function exists within the microbiome, then it would be extending its function in that microbiome^34^. As the functioning of all genes in a defined environment could not be ascertained^34^, in such case how many such conclusions stand correct? Keeping these aspects into consideration, the functionality of these metabolic pathways and protein features were assessed by metabolic fingerprinting or metabolite profiling^35^. Metabolite profiling during sodium benzoate functional assay identifies several intermediary metabolites as an outcome of sodium benzoate catabolism. Functional assay for benzoate catabolism indicates that human gut microbes quickly metabolize the available benzoate to generate metabolic intermediates in energy generation pathways. However, these metabolites were mapped only onto oxygenase-coupled protocatechuate mediated benzoate metabolism. It indicates that all metagenomic genetic machinery was not constitutively expressed. The non-functioning of the remaining pathway still needs to be explored. However, it could be either due to the limited prevalence of these gene sets or due to the non-availability of strict anaerobic conditions.

## Conclusion

The current study explores the human gut microbiome to highlight the influence of the human gut microbiome on dietary sodium benzoate and vice-versa. The current study establishes the role of the human gut microbiome in sodium benzoate catabolism, as well as indicates the various gene clusters/protein features involved in these metabolic pathways. Besides, it also enriches our understanding of the evolutionary maturation of the human gut microbiome towards changes in dietary habits and the environmental requirement for successful sustenance in the variable gut environment.

## Methodology

### Sample collection and metagenomic DNA isolation

Fecal samples (n = 8) were collected in a sterile container from healthy individuals (Age 29–36 years, Male) without any history of prolonged illness and taking proactive foods. The study was conducted after seeking ethical clearance from the human ethical committee of Maharshi Dayanand University, Rohtak, Haryana, India. Informed consent was taken before engaging the individuals in the current study. Human ethical guidelines were strictly followed before engaging individuals, as well as a written consent was taken before collecting samples. Metagenomic DNA was isolated using the alkali-lysis method^36^. The qualitative and quantitative analysis of the isolated metagenomic DNA was performed with agarose gel electrophoresis and Qubit dsDNA HS Assay Kit (Invitrogen, USA) respectively.

### Metagenome sequencing and sequence analysis

The metagenomic DNAs of the samples were sequenced with MiSeq Reagent Kits v3 using MiSeq next-generation sequencing platform (Illumina, USA) at CSIR- Institute of Genomics and Integrative Biology (IGIB), New Delhi following standard sequencing instructions (Illumina, USA). FASTQ reads were uploaded to the Metagenome Rapid Annotation using Subsystem Technology (MG-RAST) server^37^ (http://metagenomics.anl.gov/) for taxonomic and functional annotation of the sequences. Metagenome data was processed at MG-RAST^37^ for adapter trimming, denoising, and normalization, removal of sequencing artifacts, and host DNA contamination removal. Quality filtered sequences were searched for the presence of rRNA features (cutoff similarity % > 70%). Potential ribosomal RNA genes were clustered and checked for their homologs in the Ribosomal Database Project (e value < 10^–5^, sequence similarity < 60% & word size > 15 bp). FragGeneScan^38^ was employed for the identification of all putative protein-coding genes. Identified gene and their encoded proteins were described as genetic features and proteins features, respectively. Predicted protein-coding sequences were clustered (~ 90% identity) and processed for protein similarity search using the BLAT algorithm against the M5NR protein database, RefSeq database, and Subsystems database (e value < 10^–5^, minimum identity < 60%). Similarity outcome was mapped to annotate protein features of each sequence in given clusters to describe each feature abundance, Lowest common ancestor (LCA) abundance profile, Data source abundance profile to predict protein feature-based taxonomic and functional affiliation.

### Mining and characterization of benzoate metabolic gene clusters

Genetic and protein features associated with benzoate catabolism were extracted from the output of the subsystem database search analysis (e value < 10^–5^, minimum identity < 60%). Identified benzoate catabolic genetic/protein features were mapped manually onto aerobic and anaerobic benzoate metabolic pathways. Taxonomic affiliation of benzoate catabolic protein features was assessed with KAIJU webserver^39^ after searching their homologs in NCBI nr databases (e value < 0.0, minimum identity < 75%, word size > 15 bp).

### Analysis of the diverse human gut metagenomes

The human gut metagenomes of diverse populations were downloaded and analyzed to understand abundance to benzoate catabolic genetic and protein features in USA, Sweden, Venezuela, Japan, and Malaysia gut microbiomes (Supplementary Table S2). RNA and protein features were extracted and processed for their homology search in Ribosomal Database Project and Subsystem database. Benzoate catabolic (genetic and protein) features were extracted from the subsystem database search analysis. Significance testing between the metagenomic datasets was assessed using the PERMANOVA (Permutational Multivariate Analysis of Variance)^40^. Database search output was used for Principle coordinate analysis (PCoA)^40^ and heatmap^41^ analysis.

### Functional affirmation of sodium benzoate catabolism

The 200 mg of fresh human feces (n = 8) was suspended into 1 ml Phosphate buffer saline (pH 7.4). The samples were processed to collect the microbial pellet^36^. The microbial pellet was suspended in sodium benzoate solution (5 mM) followed by incubation at 37 °C. In parallel, a control sample containing microbial pellet in PBS was also incubated. Fractions were collected at different time intervals (0, 0.5, and 2 h) and centrifuged for 1 min at 13,000 rev min^−1^. Samples were quenched with 60% aqueous methanol solution (− 48 °C)^42,43^. The quenched sample was centrifuged immediately at 16060 g for 10 min at − 10 °C. The whole mixture was snap-frozen in liquid nitrogen and processed for metabolites extraction with methanol by freeze–thaw cycling^42,43^. The supernatant was collected and stored at − 86 °C after the centrifugation of the methanol suspension at 16 060 g, at − 9 °C, for 5 min. An additional amount of 500 μL of 100% methanol (− 48 °C) was added to the pellet followed by freeze–thaw cycling^42,43^. The second aliquot of the supernatant was collected again after the centrifugation of the methanol suspension at 16 060 g, at − 9 °C, for 5 min. Both supernatants were pooled and stored at − 86 °C for further analysis^42,43^. The deproteinization of samples was achieved by the addition of 400 µl acetonitrile in each vial followed by centrifugation at 5000 rev min^−1^ for 10 min. The supernatant was collected and put into an autosampler vial for injection. Unbiased metabolic profiling was performed with Exion LC system integrated AB SCIEXX500B QTOF mass spectrometer (SCIEX, USA) (Supplementary methods SM1 and SM2). LC/MS-based metabolic profiles were firstly converted into mzML data format by ProteoWizard software^44^ and sequentially handled by XCMS software running under R environment Version 3.7.1 for metabolic features detection and chromatographic matching^45^. Significant metabolic features were imported into MetaboAnalystR 2.0^46^ for the untargeted metabolic assignment of MS peaks to the metabolic pathways (Supplementary methods SM3).

## Supplementary Information

Supplementary Information

## Data Availability

Human Gut Metagenomic DNA sequences generated in this study are available at NCBI with a BioProject ID PRJNA663717 and submission ID SUB8146144.
