# Supplementary material for: Mapping of the benzoate metabolism by human gut microbiome indicates food-derived metagenome evolution
Source: Sci Rep. 2021 Mar 10;11:5561. doi: 10.1038/s41598-021-84964-6 (PMC7946887; doi:10.1038/s41598-021-84964-6)

Mapping of the benzoate metabolism by human gut microbiome indicates food-derived metagenome evolution

Monika Yadav[1], Avinash Lomash[2], Seema Kapoor[2], Rajesh Pandey[3¶], Nar Singh Chauhan[1*]

[1]Department of Biochemistry, Maharshi Dayanand University, Rohtak-124001, India

[2]Pediatrics Research & Genetic Laboratory, Maulana Azad Medical College, New Delhi-110002, India

[3]Genomics and Molecular Medicine, CSIR-Institute of Genomics and Integrative Biology (CSIR-IGIB), Delhi-110007, India

*Corresponding author

Dr N.S. Chauhan, Department of Biochemistry, Maharshi Dayanand University, Rohtak, Haryana, India (nschauhan@mdurohtak.ac.in)

(A) Supplementary methods

Supplementary method SM1: Chromatographic separation

The chromatographic separation was carried out using an ExionLC™ System fitted with an Agilent PLRP-S column (2.1 mm×50 mm, 300 Å, 5 µm) at 80°C using the gradient shown in Table 1. Mobile phase A was 0.1% formic acid in water and mobile phase B was 0.1% formic acid in acetonitrile. The column oven was set at 40 °C. Before sample analysis, column was equilibrated with the mobile phase for 30 min. Sample injection volume was 10.0 µL, and the total run time was 20 min per sample.

Table SM1. LC conditions for chromatographic separation

Time [min]	*Flow [mL/min]*	*A.Conc [%]*	*B.Conc [%]*
4.00	0.6000	95.0	5.0
8.00	0.6000	50.0	50.0
10.00	0.6000	50.0	50.0
14.00	0.6000	5.0	95.0
16.00	0.6000	5.0	95.0
18.00	0.6000	95.0	5.0
20.00	0.6000	95.0	5.0

Supplementary method SM2: Mass spectrometric detection

The AB Sciex X500B QTOF mass spectrometer was with a Turbo V Ion Source fitted with a Twin Sprayer ESI Probe was operated in the ESI- & ESI+ mode for data acquisition. Mass spectrophotometer instrument experimental conditions are listed in Table 2. Data was captured with SCIEX OS 1.4.

Table SM2. Mass spectrophotometer instrument conditions for these experiments

Parameter	Setting 1	Setting 2
Scan Mode	Positive	Negative
Gas 1	50 psi	50 psi
Gas 2	60 psi	50 psi
Curtain gas	35 psi	35 psi
Temperature	550 °C	550 °C
Ion Spray Voltage	5500 V	−4500 V
Time Bin to Sums	4	4
Accumulation Time	0.25s	0.25s
TOF Start Mass	100 da	100 da
TOF Stop Mass	2000 da	2000 da
Declustering Potential	80V	-80V
Collision Energy	10V	-10V

Supplementary method SM3: Processing of the metabolomics dataset

Metabolomics datasets were analyzed with pairwise comparative analysis with default feature detection parameters (centWave for feature detection (Δ m/z = 30 ppm, minimum peak width = 10 s, and maximum peak width = 60 s; obiwarp settings for retention-time correction (profStep = 0.5); and parameters for chromatogram alignment, including mzwid = 0.025, minfrac = 0.5, and bw = 5). The relative quantification of metabolite features was based on EIC (extracted ion chromatogram) areas (https://xcmsonline.scripps.edu/). Welch's t test was used to identify significantly different metabolic features ($p < 0.05$) presence in test (0.5, 2 hr) vs control (0Hr). The m/z value of significantly different metabolites ($p < 0.05$) were used as input in MetaboAnalystR to map them in functional meta-analysis (https://www.metaboanalyst.ca/MetaboAnalyst/ModuleView.xhtml).

B) Supplementary Tables

Supplementary Table S1. Statistics of the current human gut metagenome datasets.

	**IN1**	**IN2**	**IN3**	**IN4**	**IN5**	**IN6**	**IN7**	**IN8**
Total bp Count	1774549173	1587754523	1675963108	995200384	1,676,388,244	1,588,157,284	1471950653	1298501497
Total Sequences Count	5922623	5299189	5593588	3321517	5,593,588	5,299,188	4911443	4332698
Mean GC %					48 ± 7 %			
Artificial Duplicate Reads	1608227	1438940	1518881	901924	1291482	1223509	1133984	1000361
Post QC: bp Count	922099100	825036037	870871373	517130431	649690687	615496440	570460115	503239233
Post QC: Sequences Count	4115291	3682103	3886664	2307932	3624390	3433633	3182391	2807392
Predicted Protein Features	1878879	1681103	1774497	1053711	1580234	1497064	1387523	1224024
Predicted rRNA Features	33137	29648	31296	18584	36855	34915	32360	28549
Identified Protein Features	1119366	1001538	1057179	627763	850,620	805851	746886	658877
Identified rRNA Features	1959	1751	1850	1099	1,687	1598	1481	1309

Supplementary Table S2. Human gut metagenome datasets used for comparative analysis.

Sr No	Metagenomic Dataset	Database Id	No. of Datasets	Nature of dataset
1	USA	MGP98 (www.mg-rast.org)	59	Unassembled reads
2	Europe	MGP13068 (www.mg-rast.org)	81	Unassembled reads
3	Japan	MGP29 (www.mg-rast.org)	13	Unassembled reads
4	Malawi	MGP98 (www.mg-rast.org)	19	Unassembled reads
5	Venenzula	MGP98 (www.mg-rast.org)	21	Unassembled reads
6	Malaysia	MGP5712 (www.mg-rast.org)	8	Unassembled reads

Supplementary Table S3. Bonferroni-corrected p-values obtained after performing PERMANOVA test analysis to assess variations among gut metagenome of various populations (MA: Malawi (n=19), JP: Japan (n=13), ML: Malaysia (n=8), US: USA (n=59), VZ : Venezuela (n=21), IN: India (n=8), EU: Europe (n=81)) based on subsystem protein features.

	**MA**	**JP**	**ML**	**US**	**VZ**	**IN**	**EU**
**MA**		0.0021	0.0021	0.0021	0.5985	0.0315	0.0021
**JP**	0.0021		0.0021	1	0.3297	1	0.0021
**ML**	0.0021	0.0021		0.0021	0.0021	0.0063	0.0021
**US**	0.0021	1	0.0021		0.6363	1	0.0021
**VZ**	0.5985	0.3297	0.0021	0.6363		1	0.0021
**IN**	0.0315	1	0.0063	1	1		0.0084
**EU**	0.0021	0.0021	0.0021	0.0021	0.0021	0.0084	

Here: The H0 for PERMANOVA= centroid for 7 population is similar. The H1 for PERMANOVA= centroid for 7 population is dissimilar

Supplementary Table S4. The relative abundance of the sodium benzoate catabolic protein features across various gut metagenomes. The relative abundance was calculated with the mean value of identified sodium benzoate catabolic protein features across studied populations.

Benzoate catabolic feature	USA	Malawi	Venenzula	Japan	Malaysia	Europe
2-hydroxycyclohexanecarboxyl-CoA dehydrogenase	0	0	0.18	0	0	0.45
3-hydroxybutyryl-CoA dehydrogenase	0.26	2.3	0.53	0.93	1.09	0.51
Acetyl-CoA acetyltransferase	8.79	22.58	24.34	7.48	22.83	6.25
Benzoyl-CoA reductase subunit BadD	0.73	1.84	4.03	0	0	0.55
Benzoyl-CoA reductase subunit BadE	6.82	11.06	8.06	8.41	0	5.57
Benzoyl-CoA reductase subunit BadF	3.28	4.15	11.56	7.48	0	2.07
Benzoyl-CoA reductase subunit BadG	21.07	10.6	22.59	18.69	0	35.15
Glutaryl-CoA dehydrogenase	0.05	0	0.35	0	6.52	0.13
3-oxoadipate CoA-transferase subunit A	1.35	0	0.53	0	2.72	0.73
3-oxoadipate CoA-transferase subunit B	0.52	0	0	0.93	0	0.02
Beta-ketoadipate enol-lactone hydrolase	1.97	0.46	0.88	2.8	5.43	1.47
Catechol 1,2-dioxygenase	0.42	0	0	0	0	0
Muconate cycloisomerase	1.98	0.46	0	1.87	2.17	0.59
Muconolactone isomerase	1.2	0.46	0	0	1.09	0.07
Succinyl-CoA:3-ketoacid-coenzyme A transferase subunit A	0.26	0.92	0	2.8	26.09	0.03
Mandelate racemase/muconate lactonizing enzyme	5.93	1.38	1.58	0.93	0.54	3.16
3-carboxy-cis,cis-muconate cycloisomerase	2.24	0	0.18	1.87	5.98	0.03
4-carboxymuconolactone decarboxylase	26.01	37.33	20.67	39.25	1.09	42.06
Beta-ketoadipyl CoA thiolase	0.47	0	0	0	0	0.18
Pca regulon regulatory protein PcaR	3.23	0	0.7	0	1.09	0.02
Protocatechuate 3,4-dioxygenase alpha chain	2.5	0.46	0.53	0	1.63	0
Protocatechuate 3,4-dioxygenase beta chain	2.19	0.46	0	1.87	1.09	0
Dicarboxylic acid transporter	0	0.46	0	0.93	6.52	0
Benzoate 1,2-dioxygenase	1.93	0	0	0	3.26	0
Benzoate transport protein	4.21	4.61	2.1	1.87	2.72	0.1
Benzoylformate decarboxylase	0.1	0	0.18	0	0.54	0
Ring hydroxylating dioxygenase, alpha subunit	0.05	0	0.18	0	0	0
Benzoate MFS transporter BenK	0.68	0	0.18	1.87	3.8	0.89
Benzoate dioxygenase, ferredoxin reductase component	1.77	0.46	0.7	0	3.8	0

Supplementary Figures (C)

Supplementary Fig. S1. Phylogenetic affiliation of the human gut metagenome protein features associated with anaerobic benzoate metabolism. Krona chart (a) and bar chart (b) showing the phylogenetic hierarchy of anaerobic benzoate catabolic features at genera level and phylum level respectively.

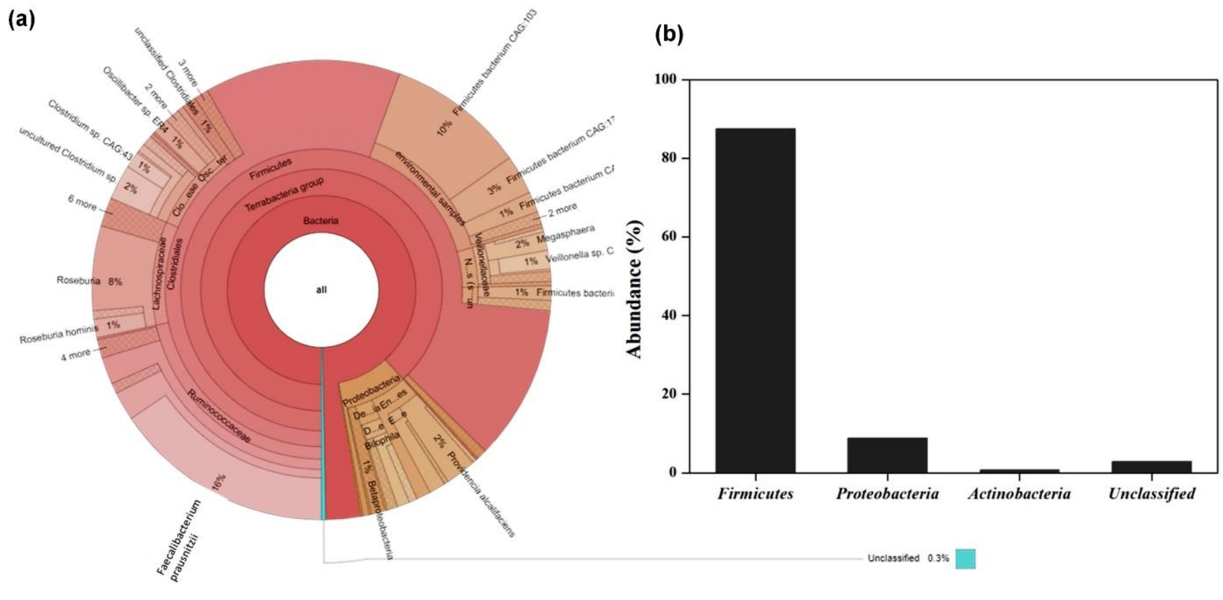

Supplementary Fig. S2. Phylogenetic affiliation of the human gut metagenome protein features associated with Catechol branch of β-ketoadipate pathway for benzoate metabolism. Krona chart (a) and bar chart (b) showing the phylogenetic hierarchy of catechol mediated aerobic benzoate catabolic features at genera level and phylum level respectively.

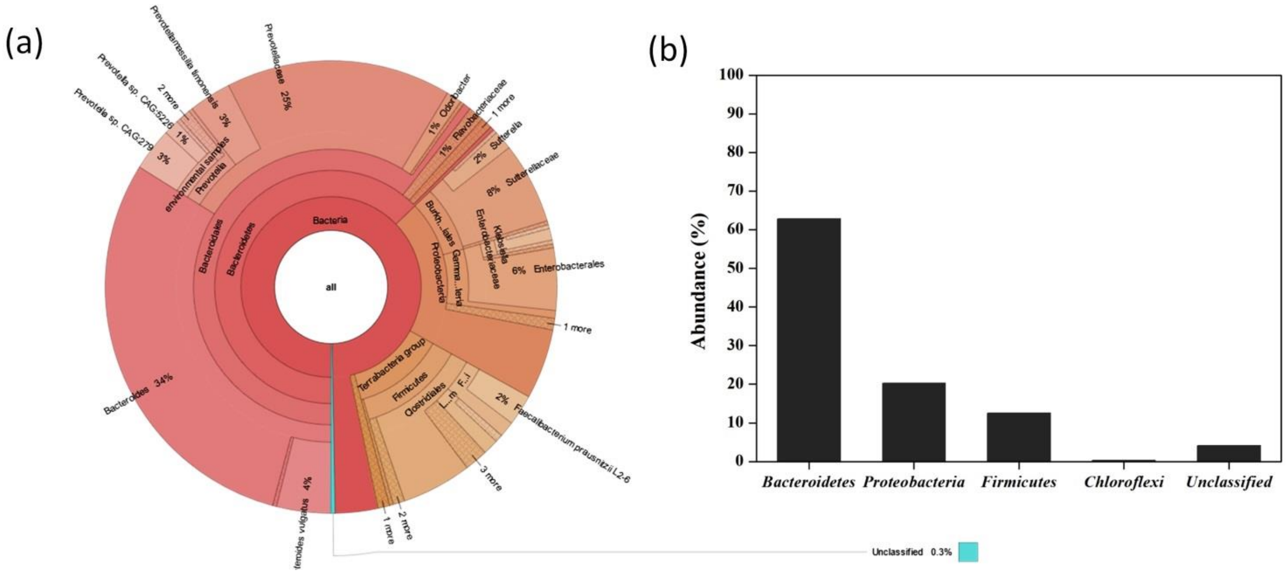

Supplementary Fig. S3. Phylogenetic affiliation of the human gut metagenome protein features associated with Protocatechuate branch of β-ketoadipate pathway for benzoate metabolism. Krona chart (a) and bar chart (b) showing the phylogenetic hierarchy of protocatechuate mediated aerobic benzoate catabolic features at genera level and phylum level respectively.

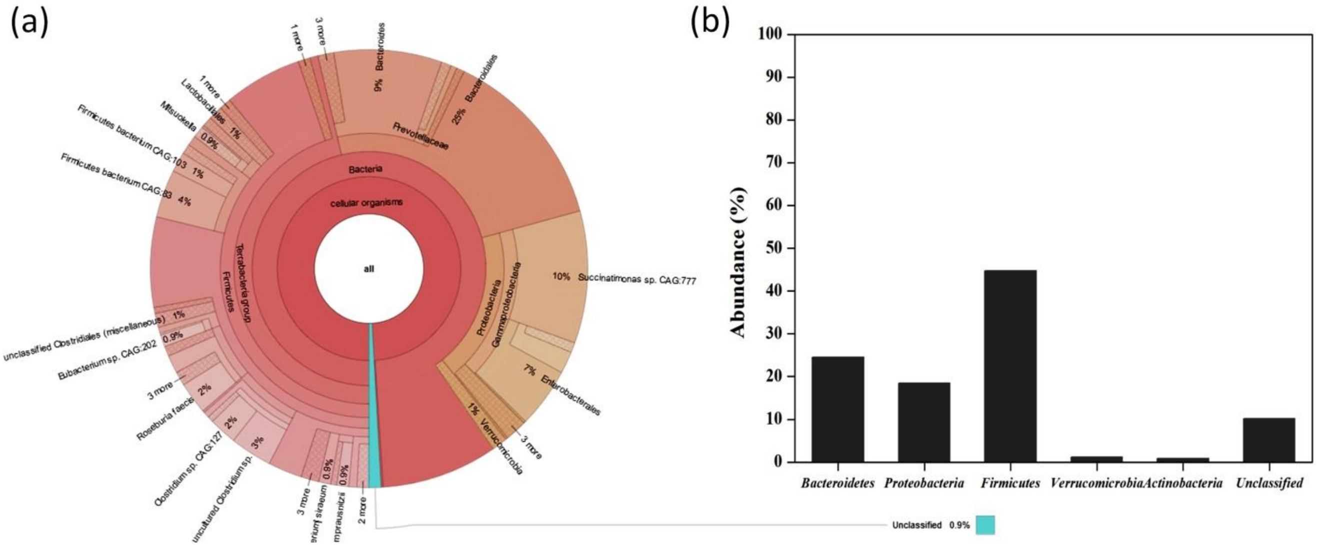

Supplementary Fig. S4. The functional assessment of sodium benzoate catabolism using LC-MS analysis.

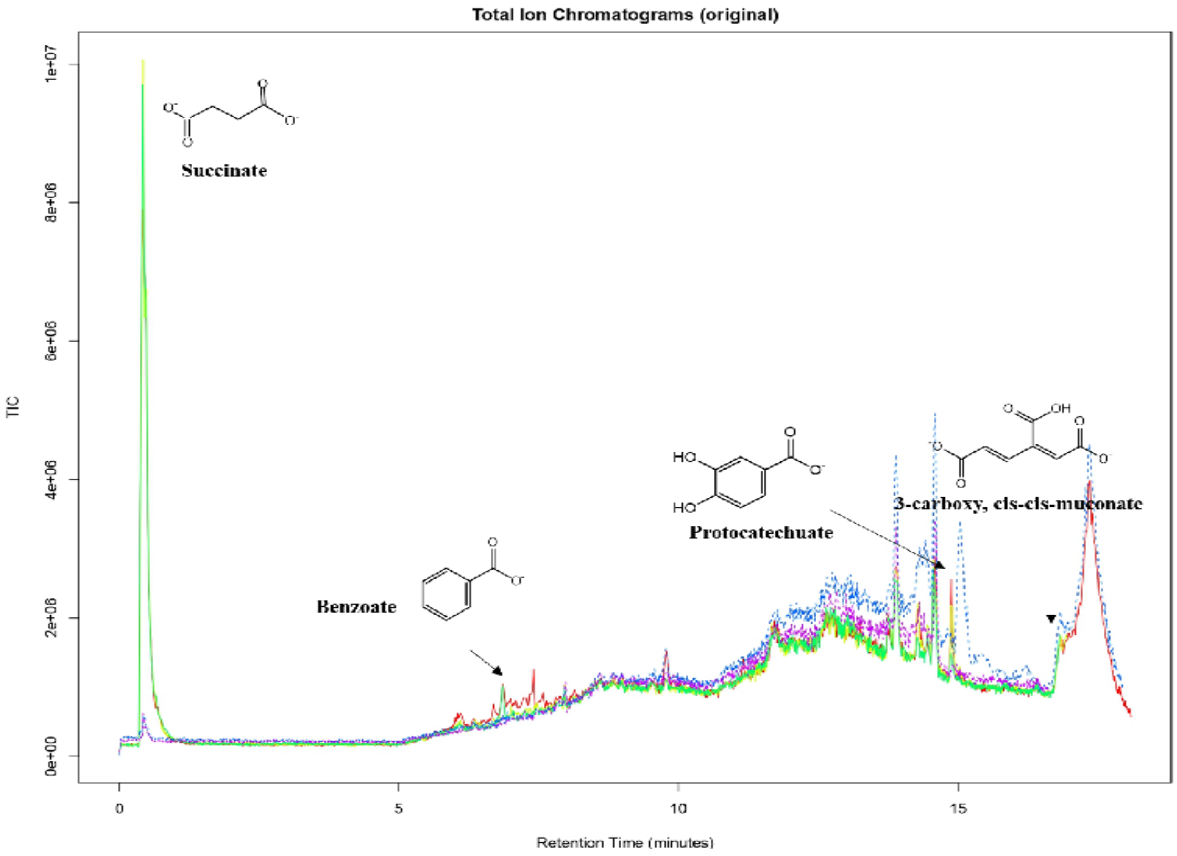

Supplement: Supplementary file 1 — Supplementary Information [file 41598_2021_84964_MOESM1_ESM.pdf]
